# Ancestry-specific recent effective population size in the Americas

**DOI:** 10.1371/journal.pgen.1007385

**Published:** 2018-05-24

**Authors:** Sharon R. Browning, Brian L. Browning, Martha L. Daviglus, Ramon A. Durazo-Arvizu, Neil Schneiderman, Robert C. Kaplan, Cathy C. Laurie

**Affiliations:** 1 Department of Biostatistics, University of Washington, Seattle, WA, United States of America; 2 Division of Medical Genetics, Department of Medicine, University of Washington, Seattle, WA, United States of America; 3 Institute for Minority Health Research, University of Illinois at Chicago, Chicago, IL, United States of America; 4 Department of Preventive Medicine and Epidemiology, Loyola University Stritch School of Medicine, Chicago, IL, United States of America; 5 Department of Psychology, University of Miami, Coral Gables, FL, United States of America; 6 Department of Epidemiology and Population Health, Albert Einstein College of Medicine, Bronx, NY, United States of America; University of California, Los Angeles, UNITED STATES

## Abstract

Populations change in size over time due to factors such as population growth, migration, bottleneck events, natural disasters, and disease. The historical effective size of a population affects the power and resolution of genetic association studies. For admixed populations, it is not only the overall effective population size that is of interest, but also the effective sizes of the component ancestral populations. We use identity by descent and local ancestry inferred from genome-wide genetic data to estimate overall and ancestry-specific effective population size during the past hundred generations for nine admixed American populations from the Hispanic Community Health Study/Study of Latinos, and for African-American and European-American populations from two US cities. In these populations, the estimated pre-admixture effective sizes of the ancestral populations vary by sampled population, suggesting that the ancestors of different sampled populations were drawn from different sub-populations. In addition, we estimate that overall effective population sizes dropped substantially in the generations immediately after the commencement of European and African immigration, reaching a minimum around 12 generations ago, but rebounded within a small number of generations afterwards. Of the populations that we considered, the population of individuals originating from Puerto Rico has the smallest bottleneck size of one thousand, while the Pittsburgh African-American population has the largest bottleneck size of two hundred thousand.

## Author summary

Using genome-wide genetic data on several hundred individuals sampled from a population, we can estimate the current effective size of the population and the changes in effective size that have occurred over the past hundred generations. Many populations in the Americas are admixed, having ancestry from Europe, Africa, and the Americas. In such cases, one can learn not only about the effective population size history of the admixed population since admixture, but also about the effective population size histories of the contributing ancestral populations. In this paper we develop methodology for estimating past effective population size and analyze data from Hispanic, African-American, and European-American populations resident in the United States. We observe differences between populations in their historical effective sizes. These differences are useful for understanding differences in disease incidence between populations and for identifying populations that will maximize power in genetic association studies.

## Introduction

Effective population size is a key factor in evolutionary genetic processes, such as drift and selection, which have important implications for medical genetics [1, 2]. With the development of agriculture, human populations have grown super-exponentially during the past few thousand years [3, 4]. More recently improved sanitation, modern medicine, and industrialized food production have further accelerated population growth. During the last few generations, growth rates have slowed or become negative in many human populations due to the availability of effective birth control methods, higher levels of education for women, urbanization, and other factors [5]. In addition to these global trends, populations in the Americas have experienced bottlenecks due to migrations, introduced diseases, and other effects of colonization.

Effective population size is a genetics-based measure of population size [6]. Here we use inbreeding effective population size defined in terms of coalescence probability (the probability that a given pair of haplotypes are descended from a single haplotype in the previous generation). Consider a population of diploid individuals. Randomly select a neutrally evolving locus and a pair of haplotypes from the population. Let *q*_*g*_ be the probability that the two haplotypes have a common ancestor *g* generations ago at that locus, conditional on not having a common ancestor in the last *g* − 1 generations. In a randomly mating population with *N*_*g*_ breeding individuals *g* generations ago (2*N*_*g*_ haplotypes), that probability would be 1/(2*N*_*g*_) because there are 2*N*_*g*_ possible ancestors for the second haplotype, each equally likely and only one of which is the ancestor of the first haplotype. Thus, solving *q*_*g*_ = 1/(2*N*_*g*_) for *N*_*g*_ we obtain the effective population size *g* generations ago as *N*_*g*_ = 1/(2*q*_*g*_). Consequently, to estimate the effective size of a population, we first estimate the conditional coalescence probability *q*_*g*_, and then use the relationship *N*_*g*_ = 1/(2*q*_*g*_). The effective size of a population is generally smaller than the census size of a population due to demographic factors such as overlapping generations [7]. In general, populations are not closed, but experience migration. Thus the effective size *g* generations ago reflects the conceptual population of ancestors that contributed to the current generation, rather than an actual population residing in a certain location *g* generations ago. Furthermore, the definition of effective size assumes random mating, but actual populations are structured by geography and by cultural factors, with preference for mating within sub-population. Thus the effective size depends on the sampling scheme, which may over-represent some of the sub-populations.

Admixed individuals have ancestry that is recently derived from more than one continental population. In the Americas, many individuals have admixed ancestry derived from indigenous peoples of the Americas, European settlers, and enslaved Africans forcibly brought to the Americas, as well as more recent immigration. Because the migration events that brought these continental groups together are recent (less than 20 generations before present in the Americas), the chromosomal segments of single-continent ancestry tend to be long, and it is possible to infer the ancestry at most points in the genome from genotype data [8–11]. Analyses can then be performed using only the parts of the genome that are inferred as derived from a particular ancestry. This enables inference about the ancestral populations. An example of ancestry-specific analysis of admixed data is ancestry-specific principal components analysis, which can be used to investigate the degree of genetic similarity between the ancestors of individuals in an admixed sample and present-day individuals in a geographical region [12, 13].

When considering the effective size of an admixed population, the overall effective size of the population is generally the most relevant quantity subsequent to admixture. In contrast, preceding admixture the overall effective population size of the combined contributing ancestral populations may not be the quantity of interest. Instead, one may wish to know the historical effective sizes of those individual ancestral populations. Consider two haplotypes of a particular ancestry that are sampled from the admixed population at a fixed locus. Looking back in time, if those two haplotypes have not coalesced at this locus by the time of the onset of admixture, the probability that they coalesce in the previous generation depends (as noted above) on the effective size of the population in which the haplotypes were located at that time, which is the particular source ancestral population. For example, in a sample of admixed individuals from a Latin American location, the ancestral populations may be from Europe (primarily Spain), Africa (primarily West Africa), and America. The ancestral American populations are primarily those that were resident pre-admixture in the region around the sampling location, but can include a broader region if the sampling location is home to significant numbers of migrants from elsewhere in Latin America. When we calculate ancestry-specific effective population size for pre-admixture times, we are estimating the effective sizes of the source populations that participated in the admixture, which could be subsets of larger populations (e.g. Spanish colonizers within the larger Spanish population). Although we can also calculate ancestry-specific effective sizes post-admixture, these may not be particularly meaningful because they do not correspond to any actual population of individuals.

Identity-by-descent (IBD) sharing in population samples can be used to estimate recent effective population size [14, 15]. Segments of IBD can be detected in genotype data. We consider segments with genetic length greater than 2 centiMorgans (cM). These segments are due to inheritance from recent common ancestors within the past several hundred generations. The numbers and lengths of IBD segments contain information about coalescence probabilities. Shorter segments of IBD represent coalescent events that occurred further back in time (up to several hundred generations ago), while longer segments represent coalescent events that occurred in the past few generations. If the number of IBD segments is high, a larger number of coalescence events have occurred, indicating that the coalescence probability is high and thus the effective size is low. Similarly, if the number of IBD segments is low, the effective size is high. These relationships can be quantified mathematically for estimation of effective size, including estimation of changes in effective population size over time.

A previous study estimated ancestry-specific historical effective population size using the site frequency spectrum of alleles in ancestry segments from admixed individuals [16]. The site frequency spectrum interrogates a much more distant time period than the IBD-based method that we use here. Site frequency spectrum methods also require sequence data, whereas our IBD-based method can use single nucleotide polymorphism (SNP) array data, increasing the range of existing data to which it can be applied.

We demonstrate the effectiveness of our ancestry-specific effective population size estimation methodology with simulated data, and then use our methodology to estimate ancestry-specific recent effective population size in populations from the Hispanic Community Health Study/Study of Latinos (HCHS/SOL), and in African-American and European-American populations in two US cities from the Healthy Aging and Body Composition (Health ABC) study.

## Results

### The IBDNe method

We first summarize the key points of the IBDNe method for estimating the effective population size of homogeneous populations (further details may be found in [14]), and then describe how this method can be applied to ancestry-specific effective size in admixed populations.

Consider a population with an effective size of *N*_*g*_ diploid individuals *g* generations before the present (generation 0 is the current generation, generation 1 is the most recent previous generation, and so on). If *q*_*g*_ is the probability that a pair of haplotypes randomly sampled from the current generation coalesce (have most recent common ancestor) at generation *g* given that they have not coalesced by generation *g* − 1, then *N*_*g*_ = 1/(2*q*_*g*_) by definition (see Introduction). Suppose we have a sample of individuals from the current generation, and we identify long segments of identity by descent between pairs of haplotypes drawn from different individuals, finding all those segments of identity by descent that exceed some length threshold *C*. In most settings, we use *C* = 2 cM, since we have high power to detect such segments using existing methods such as Refined IBD [17]. Then, if the past trajectory of effective size ***N*** = {*N*_*g*_: *g* ≥ 1} was known, we could calculate *E*_*g*_, the expected total length of detected IBD genome-wide that is attributable to most recent common ancestry at generation *g*. Also, if the effective size trajectory ***N*** was known, we could calculate the probability that an IBD segment of length *l* is due to most recent common ancestry at generation *g*, and hence obtain *O*_*g*_, the expected total length of detected IBD genome-wide that is attributable to most recent ancestry at generation *g*, conditional on the observed IBD segments. Both *E*_*g*_ and *O*_*g*_ are functions of ***N***. Finding values of *N*_*g*_ that give equal values of *E*_*g*_ and *O*_*g*_ provides a methods of moments estimate of ***N***. We iterate estimating ***N*** and re-calculating *E*_*g*_ and *O*_*g*_ until convergence of our estimate of ***N***. During the iterative estimation process we also impose a smoothness requirement on *N*_*g*_ as a function of *g* to aid in the estimation.

Now consider an admixed population, in which local ancestry has been determined. The ancestry-specific effective size $N_{g}^{\left(a\right)}$ for ancestry *a* considers only those haplotypes that are descended from ancestry *a*. If $q_{g}^{\left(a\right)}$ is the probability that a pair of such haplotypes randomly sampled from the current generation coalesce at generation *g* given that they have not coalesced in generations 1 to *g* − 1, then $N_{g}^{\left(a\right)}=1/(2q_{g}^{\left(a\right)})$. For generations prior to the admixture event, this ancestry-specific effective size $N_{g}^{\left(a\right)}$ represents the total effective size of the ancestral population that contributed to ancestry *a* in the admixed population. For example, if considering European ancestry, and the European ancestors came from some population in Spain, the pre-admixture European-specific effective population size will be the effective size of that population in Spain. Our interest in the ancestry-specific effective population size is mainly for the pre-admixture effective population sizes, but we also obtain estimates of post-admixture ancestry-specific effective population sizes. If the post-admixture population is randomly mating, and has proportion *p*^(*a*)^ of its ancestry being of ancestry *a*, then it is straightforward to show that $N_{g}^{\left(a\right)}=p^{\left(a\right)}N_{g}$ where *N*_*g*_ is the overall effective size of the admixed population. If there is assortative mating or ongoing migration, this relationship will not hold. We now discuss how to estimate $N^{\left(a\right)}=\{N_{g}^{\left(a\right)}:g\geq 1\}$ using the IBDNe framework.

The IBDNe framework needs the following information: the IBD lengths, in order to obtain *O*_*g*_; and the number of pairs of sampled haplotypes, which are needed to obtain *E*_*g*_. With a homogenous population, we obtain the IBD lengths directly from the detected IBD segments, and we calculate the number of pairs of sampled haplotypes from the number of sampled individuals. With ancestry-specific analysis, there are differences because we are only interested in IBD between haplotypes of the given ancestry, but ancestry is not constant along the genome. This affects both the way in which the IBD lengths are handled, and the way in which the number of pairs of sampled haplotypes is calculated. Some IBD segments in an admixed population will have a very recent common ancestor (from the generations post-admixture), and since this ancestor is admixed, the IBD segment may include more than one ancestry. Only those parts of the segment that are derived from the ancestry of interest will contribute to the total length of detected IBD for this ancestry, but we still need to know the length of the whole IBD segment in order to calculate the probability that the most recent common ancestor lived in generation *g* (see Methods). Further, the number of pairs of sampled haplotypes of the given ancestry now varies from one genomic position to another, because the ancestry of each individual’s DNA varies along the genome, but the expected number of pairs of sampled haplotypes of the given ancestry can be calculated from the genome-wide ancestry proportions (see Methods). Apart from these differences, estimation of ancestry-specific effective population size is the same as for estimation of overall effective population size, and the existing IBDNe software may be used. An example analysis pipeline is provided at http://faculty.washington.edu/sguy/asibdne/.

### Accuracy in estimating ancestry specific effective population size

We simulated an admixed population with ancestry from three continental groups in order to test the accuracy of our methods. The simulation includes a low rate of genotype error (0.1%) and the simulated data have a marker density that is similar to a 1M-feature SNP array. A full description of the data simulation can be found in Methods (in the “Simulated data” section). Briefly, we used a coalescent-based simulator to simulate the Africa-Europe-Asia demographic history estimated by the 1000 Genomes Project [18], and added population bottlenecks and admixture occurring 12 generations ago.

**Fig 1:**
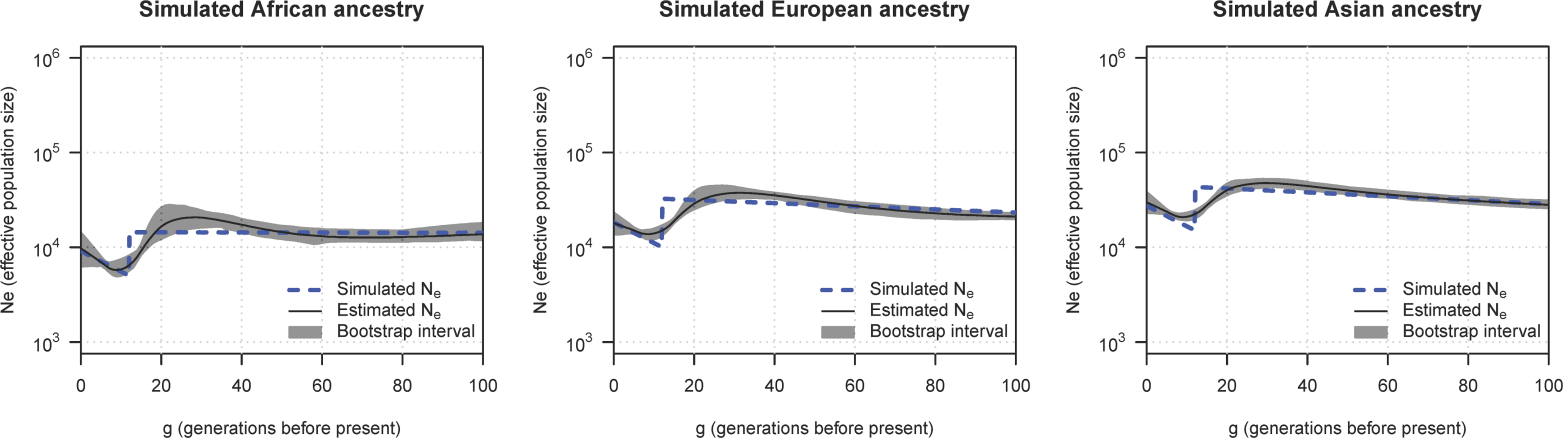
Estimated ancestry-specific effective population size in simulated data. Analysis of 500 simulated individuals from a three-way admixed population. Each column is one of the three simulated ancestries. The y-axes show ancestry-specific effective population size (*N*_*e*_), plotted on a log scale. The x-axes show generations before present. The dashed lines show simulated effective population sizes. The solid black lines show estimated ancestry-specific effective population sizes, and the gray regions show 95% bootstrap confidence intervals.

Fig 1 shows true and estimated ancestry-specific effective population size. We see that important aspects of the effective population size trajectory are represented in the estimated trajectories, including the approximate timing of the population bottleneck, the approximate size of the ancestral population, and the effective size of the ancestry-specific fraction of the admixed population after the bottleneck. The bootstrap confidence intervals do not always cover the true value, but they provide an approximate measure of the precision of the estimates.

In order to keep the simulation from becoming overly complex, the simulated population size decrease associated with migration occurs instantaneously, resulting in a sharp bottleneck in population size. The effective population size estimation procedure cannot fully capture this sharp change because it applies smoothing by fitting exponential growth curves to groups of 8 generations. In real life admixture events, such as that associated with the colonization of the Americas, we would not expect population changes to occur instantaneously. Rather, migration and population decline would have taken place over the course of several generations, resulting in smoother effective size trajectories.

We simulated two admixture scenarios that don’t include bottlenecks. One has the three populations merging without population size reductions, while the other has continuous migration from two of the populations into the third. We estimated ancestry specific effective population sizes and show the results for the merging scenario in S1 Fig and for the continuous migration scenario in S2 Fig. Because these scenarios don’t include large population size changes over short time intervals, the estimates closely match the underlying true values, and the bootstrap intervals mostly cover the true values.

We also simulated an admixture scenario with recent population structure, with or without biased sampling across the sub-populations, and show the results in S3 Fig. With unbiased sampling the results are similar to those for the main simulation without structure (Fig 1), while with biased sampling the ancestry-specific effective sizes are underestimated in the first few generations.

### Ancestry-specific effective population size in the HCHS/SOL populations

**Fig 2:**
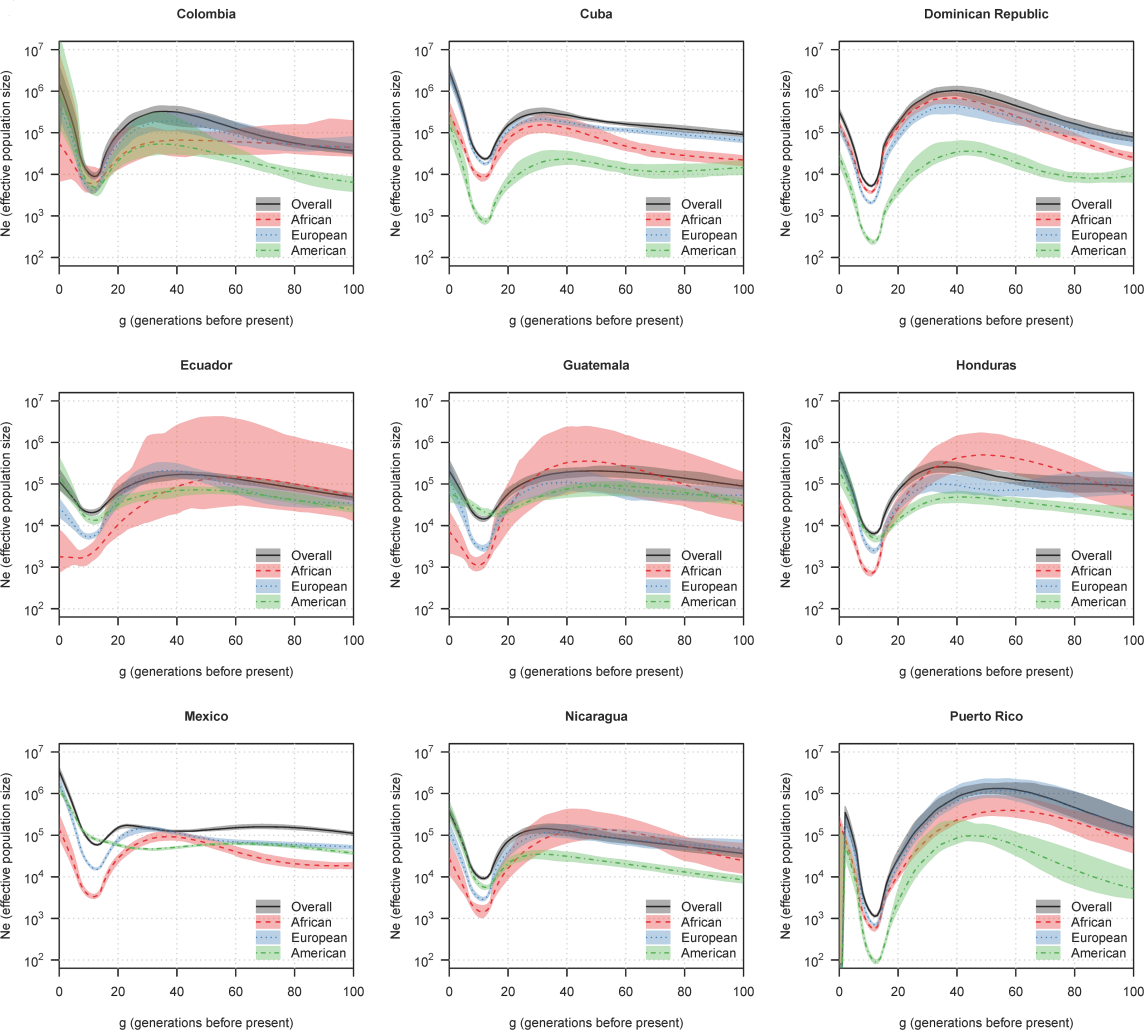
Estimated ancestry-specific effective population size in HCHS/SOL data. The y-axes show ancestry-specific effective population size (*N*_*e*_), plotted on a log scale. The x-axes show generations before the present. The lines show estimated ancestry-specific effective population sizes, and the colored regions show 95% bootstrap confidence intervals.

We group individuals in HCHS/SOL into populations based on the reported country-of-origin of their grandparents. Individuals with missing grandparental origins or grandparents from different countries are omitted from the analysis. Fig 2 shows estimated ancestry-specific effective population sizes and overall effective population sizes for the HCHS/SOL populations for the past 100 generations. S1 Table shows average total length of detected IBD segments shared by unrelated pairs of samples, which is a summary of the data used to estimate the overall effective population size. Table 1 gives sample sizes for each population. When the overall sample size is low, the amount of data for estimating the overall effective population size is low and the estimates will have a high level of uncertainty. Similarly, when the average genome-wide ancestry proportion multiplied by the sample size is low, the amount of data for estimating the ancestry-specific effective population size is low. The bootstrap intervals give approximate measures of precision of the estimates. Estimates with wide intervals should be disregarded because the bootstrap intervals may not capture the full extent of the uncertainty in these estimates. The widest intervals occur when the sample size and/or average ancestry proportion is lowest. Wide pre-bottleneck confidence intervals are also seen for Puerto Rico, despite its high sample size, due to the extremely small bottleneck that occurred in this population. The small bottleneck means that many of the possible coalescences between haplotypes occurred in the post-bottleneck period, leaving few independent haplotypes to provide information about the pre-bottleneck period. In consequence, we recommend against drawing conclusions about the pre-bottleneck sizes of the populations ancestral to Puerto Ricans from this analysis.

**Table 1:** HCHS/SOL populations.

Population^a^	Number of individuals
Colombia	184
Cuba	1301
Dominican Republic	1016
Ecuador	243
Guatemala	217
Honduras	271
Mexico	3589
Nicaragua	378
Puerto Rico	1644

^a^Individuals in HCHC/SOL with all four grandparents having reported origin from the specified country.

The results for Puerto Rico show an apparent severe drop in overall effective population size in the most recent couple of generations. This is an artefact resulting from an excess of relatives in the Puerto Rican sample, particularly at the level of 2^nd^ to 3^rd^ cousins. Although we exclude IBD from relatives that are half-siblings or closer (including parent-offspring pairs and full siblings), it is not straightforward to exclude IBD from more distant relatives without the use of pedigree information, and the IBDNe program is not designed to allow for the removal of these more distant relatives. Inclusion of these relatives in the analysis results in an excess of long IBD segments which depresses the estimate of effective population size in the last few generations. This effect may also somewhat depress estimates of effective population size in the last few generations in the other HCHS/SOL populations, though clearly not to such an extreme as for the Puerto Rican sample.

Most of the populations and ancestries show a clear population bottleneck around 12 generations ago. Colonization began earlier, around 17 generations ago (approximately 500 years ago, assuming 30 years per generation), but occurred over the course of multiple generations. Overall bottleneck sizes vary considerably across the populations, ranging from 1,000 for Puerto Rico to 60,000 for Mexico. Growth in overall effective population size subsequent to the bottleneck is estimated to have been very high in all the populations, with estimated current effective sizes in the hundreds of thousands or millions (Table 2).

**Table 2:** Maximum and minimum estimated effective population sizes (95% confidence intervals, in thousands).

Population^a^	Current size^b^	Bottleneck size^c^	American bottleneck size^d^	American pre-admixture size^e^
Colombia	58–3930	8–11	3–7	31–306
Cuba	2260–4290	22–25	0.6–0.9	18–36
Dominican Republic	270–381	5.0–5.5	0.21–0.25	30–63
Ecuador	76–224	18–24	11–16	58–84
Guatemala	126–364	13–16	16–23	53–113
Honduras	338–608	6.0–6.9	4.1–5.5	36–66
Mexico	2980–4210	57–61	58–66	84–95
Nicaragua	316–490	8.6–9.7	5.0–6.3	28–43
Puerto Rico	213–490	1.1–1.2	0.08–0.10	74–197
Memphis AA	446–814	106–139		
Memphis EA	8780–20000	126–152		
Pittsburgh AA	696–1920	164–224		
Pittsburgh EA	527–2830	17–19		

^a^ Population is country of origin of grandparents for the HCHS/SOL populations (first nine populations in table). For the Health ABC populations (last four populations in table), EA is European American, AA is African American.

^b^ All estimated effective population sizes are given in thousands. Current size is the maximum estimated overall effective size in generations 0–9, to allow for apparent effective size decreases in the last few generations due to relatives in the sample. 95% bootstrap confidence intervals are given.

^c^ Bottleneck size is the minimum estimated overall effective size in generations 7–19, except Pittsburgh EA where it is the minimum in generations 7–29 due to the earlier bottleneck time in that population.

^d^ Minimum American-specific estimated effective size in generations 7–19, to represent the bottleneck size of the ancestral American population.

^e^ Maximum American-specific estimated effective size in generations 11–99, to represent the pre-admixture effective population size.

When interpreting the drop in ancestry-specific effective population size at colonization, one must consider population structure. Populations are not closed units, since there is always migration between regions. When one considers the effective size of a “population”, one is considering the effective number of ancestors of the individuals in that population. For example, if the population is a village in a region with low migration, most of the parents and grandparents (corresponding to effective size at generations 1 and 2, respectively) will be derived from that village and the effective size will reflect the effective size of the village. However, when one looks back 20 generations, many of the ancestors may have come from nearby villages, and the effective size will reflect the effective size of the region containing those villages. Thus the effective size for the village may be lower in recent generations than in more distant generations, even if the census population size in the village and region has been stable. This effect was noted in a previous IBD-based analysis [15]. In the context of HCHS/SOL, the effective size 20 or so generations before admixture may reflect larger regional effective sizes while the immediate pre-bottleneck sizes reflect smaller sub-populations. Thus the changes in size between the maximum pre-admixture effective population size and the bottleneck effective population size (Fig 2 and Table 2) reflect these population structure effects as well as the effects of colonization.

**Fig 3:**
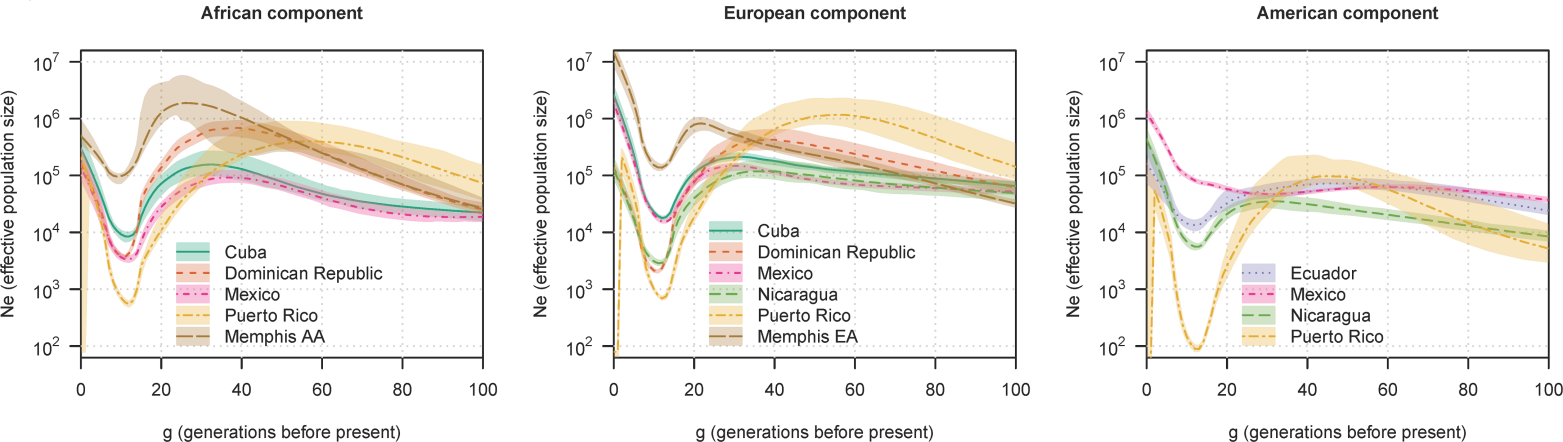
Ancestry-specific effective population size for selected populations. The y-axes show ancestry-specific effective population size (*N*_*e*_), plotted on a log scale. The x-axes show generations before present. The lines show estimated ancestry-specific effective population sizes, and the colored regions show 95% bootstrap confidence intervals. Each plot shows a different ancestral component. HCHS/SOL populations are included if the sample size multiplied by the average genome-wide ancestry proportion for the given ancestry in that population is at least 100. African ancestry for African American (AA) in Memphis, and European ancestry for European American (EA) in Memphis are included for comparison.

Fig 3 shows selected population-ancestry combinations for which the precision is relatively high. African-American and European-American populations from Memphis are included for comparison. Considering each ancestry in turn, we see similarities and differences between populations in the estimated pre-admixture effective population sizes. In the African component, we see smaller estimated pre-admixture effective sizes for Cuba (150,000) and Mexico (100,000) than for the Dominican Republic (700,000), suggesting that the African ancestors of the former two populations came from smaller sub-populations of Africa than the African ancestors of the latter two populations. In the European component we see smaller estimated pre-admixture effective sizes for Cuba (200,000), Mexico (150,000), and Nicaragua (120,000) than for the Dominican Republic (400,000). In the American ancestral component, the estimated pre-admixture effective sizes are similar between Nicaragua (400,000), Ecuador (700,000), and Mexico (600,000).

We can also look at the relative magnitude of the bottleneck population sizes to the pre-admixture sizes, bearing in mind the potential effects of population structure discussed above. For the African and European ancestral components, the drops in size are presumably mostly related to founder effects induced by migration. In contrast, for the American ancestral components, the drops in effective population size are likely due to the negative impacts of colonization including war and disease. Mexico had a relatively smaller estimated reduction in American-specific effective population size compared to the other populations (Fig 3 and Table 2).

In interpreting these results, it is important to recognize that the sampled individuals were residents of four major cities in the United States. Thus the results apply to those particular urban populations, and not necessarily to the entire countries-of-origin represented. If the populations in the US are derived from regional subsets of the countries-of-origin, the estimated effective sizes will be smaller than would be found for the countries as a whole if one had samples of individuals drawn randomly from those countries. This would be expected to have a significant influence on the estimated effective size of the most recent generations, and less influence on more distant generations due to mixing within the population over time.

### Ancestry-specific effective population sizes in the Health ABC populations

In order to further investigate the demographic history of US populations we analyzed data from the Health ABC study, which is comprised of samples from Memphis and Pittsburg (Table 3). As in the HCHS/SOL populations, the Health ABC populations show significant growth in the most recent generations, as expected. The overall bottleneck effective sizes for the Memphis populations and for the Pittsburgh African-American population are 130,000–190,000 which is more than twice as large as those for any of the HCHS/SOL populations. The pre-admixture African-specific effective population sizes for the Memphis and Pittsburg populations are 1–2 million, and thus are higher than those for HCHS/SOL populations (Fig 3). The pre-admixture European-specific effective sizes for the African-American populations and for the Memphis European-American population are around 1 million, and thus are also higher than those for most of the HCHS/SOL populations.

**Table 3:** Health ABC populations.

Population	Number of individuals
African American in Memphis	551
African American in Pittsburgh	588
European American in Memphis	863
European American in Pittsburgh	801

**Fig 4:**
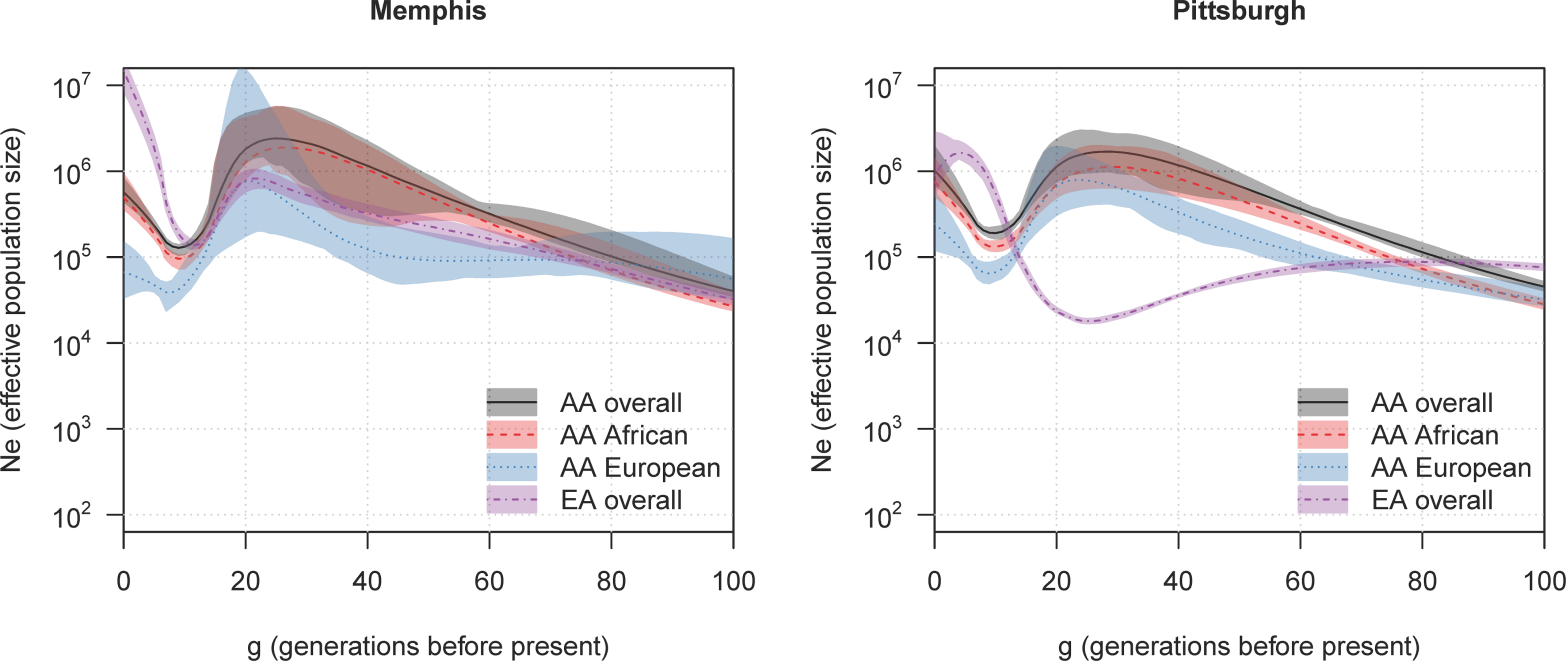
Estimated effective population size in two US cities. The y-axes show ancestry-specific effective population size (*N*_*e*_), plotted on a log scale. The x-axes show generations before present. The solid lines show estimated effective population sizes, and the colored regions show 95% bootstrap confidence intervals. Overall effective sizes are shown for the African American (AA) and European American (EA) populations, as well as ancestry-specific effective sizes for African and European ancestry in the African-American populations.

The estimated ancestry-specific effective population sizes for the African-American populations in these two cities are similar to each other (Fig 4). The similarity of the estimated demographic histories of the Memphis and Pittsburgh African-American populations suggests significant historical mixing within the larger African ancestry population that encompasses these cities, so that the two populations have a shared demographic history. In particular, the similarity of the estimated demographic histories of the European ancestry component is consistent with previous analysis of genetic data from African Americans [19], which indicates that most of the European admixture in US African American populations occurred in the southern US prior to the Great Migration movement of African Americans from the South to elsewhere in the US.

In contrast, the demographic histories of the European-American populations in Memphis and Pittsburgh differ, both before and after the founding bottleneck. Prior to the founding bottleneck, the effective population size of the Memphis European-American population was similar to that of the European component of the two African-American populations, suggesting that the European ancestors of these populations were drawn from the same European source, which again is consistent with the European admixture in the African-American populations having occurred in the South. Memphis European-Americans have a higher estimated current effective size than Pittsburgh European-Americans, which contrasts with a previous report of more long segments of IBD between European Americans in the South than in the Northeast [19]. Urban areas often differ from the general population in having more immigrants, both domestic and foreign. During the period 1910–1950, Memphis grew rapidly, tripling in size from 130 thousand to 400 thousand, while Pittsburgh’s population only increased slowly, from 530 thousand to 680 thousand [20]. Since 1950, Memphis’s population has continued to grow, while Pittsburgh’s population has declined. Thus, it is likely that Memphis’s European American Health ABC population has more diverse geographical origins on average than Pittsburgh’s European American Health ABC population, leading to the larger current effective population size.

A striking difference between the two demographic histories is that the estimated bottleneck size in Pittsburg European-Americans is significantly smaller than that in Memphis, and the timing of the bottleneck appears to be earlier (> 20 generations ago versus around 10 generations ago), which is earlier than the timing of European migration to North America. Also the decrease in population size approaching the bottleneck is very gradual, which suggests that the ancestors came from sub-populations that had quite slow rates of mixing with the broader European ancestral population. One possible explanation that could fit both of these characteristics is that many of the ancestors of the European-American population in Pittsburgh may have been members of groups that formed relatively small separated populations within Europe prior to their migration to the US. An example of such an ancestral group that fits the historical record would be the Anabaptists (including Mennonites and Amish), who separated from other European populations around 500 years ago and migrated to the US in large numbers to escape religious persecution. Many of these Anabaptists settled in Pennsylvania [21]. Although this is one possible explanation, it is not the only possibility, and our data do not address the question of origins.

## Discussion

In this paper we presented a method for calculating ancestry-specific recent effective population size by integrating local ancestry calls with inferred IBD segments. With this approach, one can estimate the demographic history over the past several thousand years of populations ancestral to current-day admixed populations. We applied our method to data from admixed populations sampled in the United States.

Our method is based on iterated method of moments. As such, it is not guaranteed to make full use of all information contained in the data, although we show that it is able to make accurate inferences from moderately large samples. One source of information that we do not incorporate in our method is the observation that any IBD segment that contains a switch in ancestry must necessarily be due to a post-admixture ancestor. This information could improve the estimation of the number of generations to the most recent common ancestor of the IBD segment if the time of the onset of admixture is known. However only a small proportion of IBD segments would provide this additional information because most IBD segments will not have a switch in ancestry and could be inherited from either pre- or post-admixture ancestors.

Previous methods for ancestry-specific effective population size estimation have not been able to estimate changes in effective size in the recent past, so the approach presented here opens new avenues for inference of demographic history. Whereas Gravel et al. [16] estimate constant American-specific effective population sizes over the past ~12,000 years, our method allows for estimation of population growth and bottlenecks during the past 500 years. Our estimates (Table 2 and Fig 2) of the American-specific effective size of Mexico are fairly flat over the past 500 years, and are in agreement with Gravel et al.’s estimate of 62,000. We estimate an order of magnitude bottleneck around 13 generations ago for Colombia’s American ancestry population, and a three orders of magnitude bottleneck around 13 generations ago for Puerto Rico’s American ancestry population. The estimates of American-specific effective size given by Gravel et al. (7,000 for Colombia and 2,000 for Puerto Rico) are intermediate between our bottleneck and maximal pre-admixture estimated sizes.

A caveat of our approach, and that of other methods based on local ancestry calls, including that of Gravel et al. [16], is that it depends on the accuracy of the local ancestry calls and inferred IBD segments. In simulated data that was designed to have similar characteristics to real human data, the results produced with our analysis pipeline had good accuracy, giving confidence that the results presented here are sound.

Our approach requires at least a few hundred samples. Additional samples are required when considering an ancestral component that forms a relatively small proportion, *p*, of the overall ancestry of the population. For most human populations, we recommend the use of sample sizes, *n*, that are sufficiently large so that *np* > 100. The precision of the estimates depends on the total number of IBD segments detected, which depends not only on *np* but also on the effective population size that is being estimated, so that a larger sample size will be needed in populations of larger effective size. In addition, in populations with extremely small bottleneck sizes the pre-bottleneck estimates will have low precision due to a high proportion of haplotypes coalescing around the time of the bottleneck, leaving few haplotypes to provide information about the pre-bottleneck sizes. When the sample size is small, or when considering effective sizes prior to a strong bottleneck event, the bootstrap intervals are larger, indicating lack of precision. In our experience, when the bootstrap intervals are particularly large they have significantly lower coverage than the nominal 95% and hence the results may be unreliable. For this reason, we suggest avoiding making inferences about the apparently large pre-bottleneck sizes of the populations ancestral to Puerto Ricans.

Our method estimates effective population size, which provides much information about population history including the approximate timing of population bottlenecks associated with migration and colonization. Admixture likely began at approximately the same time as the bottlenecks, however our method does not assume this or prove it to be the case. Other approaches can be used to directly estimate admixture times and other aspects of population history such as specific source populations [12, 16, 22].

We can compare our results to historical records, when those are available. The Trans-Atlantic Slave Trade Database (www.slavevoyages.org) provides numbers of disembarked slaves in different regions of the Americas. The total number of slaves disembarked in Mainland North America was 303,209 which is two to three times as high as the estimated bottleneck African-specific sizes in Memphis and Pittsburgh. The number of slaves disembarked in Puerto Rico was 23,779 while the estimated bottleneck size was 540; 728,809 slaves disembarked in Cuba while the estimated bottleneck size was 8400; 28,818 slaves disembarked in Hispaniola while the estimated bottleneck size in the Dominican Republic was 3700. Thus the estimated African-specific bottleneck effective population sizes are all much smaller than the numbers of arriving slaves. Several factors may play a role in this: first, many slaves died soon after arrival in the New World; second the sex-ratio in the slave trade was imbalanced with around 60% of imported slaves being male [23]; third, there are factors of geographically-based population structure involved, in that not all slaves disembarking at a regional port would have stayed in that region to contribute to the ancestry of the current-day population of that region.

In modern human populations with low levels of immigration, the overall effective population size tends to be around one-third of the census size, largely because the generation time is approximately one-third of the expected lifespan [24]. In a previous analyses of the European-ancestry populations of the UK and Finland, the recent estimated effective sizes were in line with this expectation [14]. In contrast, in this study, we did not find that estimates of overall recent effective size were around one-third of the census values. There are several reasons for this. First, in the case of Pittsburgh and Memphis, the populations are cities and experience large amounts of migration, including immigration from other regions of the US. Thus it is not surprising that the estimated current effective sizes (Table 2) are significantly larger than the census sizes of these cities (less than 1 million in each case, although the populations of the broader metropolitan areas are significantly larger). Second, in the HCHS/SOL analysis, the individuals sampled are far from being a random sample of individuals from their respective countries of origin: they are immigrants to the US, and they were sampled by household in four US communities. The household design results in an excess of relatives in some of the populations, which reduces most recent effective size estimates. The restriction to immigrants and then further to four US communities also reduces post-immigration effective population sizes. Finally, immigrants are a self-selected group that is unlikely to be representative of the country-of-origin as a whole, and will likely have over-representation from certain sub-populations. Our simulations showed that biased sampling of a structured population results in underestimation of most recent effective population size. When we compare the estimated current effective sizes of HCHS/SOL country-of-origin populations to World Bank population sizes (accessed via Google Public Data Explorer) from 1995 (when the average age of the sampled individuals was around 25), we find that the ratio of current estimated effective size to 1995 population size ranges from approximately 1/60 (Ecuador) to approximately 1/4 (Cuba), with typical values around 1/10. Although estimates of effective size in the most recent generations are affected by these issues, our simulations also showed that less recent generations are not affected. Thus our estimates are useful for learning about the effective population sizes at and before admixture.

We found that the overall bottleneck (founder) effective population size for the population of individuals of Puerto Rican origin was significantly lower than for the other populations considered. Populations with small founder sizes can have medically important genetic variants at moderate frequencies that would be extremely rare elsewhere in the world [2, 25]. Such populations are valued for genetic studies, because relatively small samples from such populations are often sufficient to determine the locations of disease-associated variants.

In analysis of data from two US cities (Memphis and Pittsburgh), we found that the estimated historical effective sizes of the African-American populations from the two cities were very similar, consistent with a shared demographic history. In contrast, the estimated demographic histories of the European-American population in each city were different.

In conclusion, estimates of ancestry-specific recent effective population size can shed light on past demographic events and suggest directions for future medical genetics research. A caveat for the results presented in this paper is that they apply specifically to the sampled populations. The HCHS/SOL individuals were sampled in four major US cities, and may not be representative of their countries of origin or of Hispanics throughout the US. The individuals sampled in the Health ABC study were at least 70 years old when they were recruited in 1997–1998, and thus they are not necessarily representative of current-day individuals living in Memphis and Pittsburgh.

## Methods

### Simulated data

We used msprime [26] to simulate three continental populations (Africa, Europe and Asia) with recent admixture. Our pre-admixture model is based on a published model inferred from the 1000 Genomes project data [18]. The initial population size (in Africa) was 7310, which increased 5920 generations ago to 14474. The out of Africa event occurred 2040 generations ago, with the out-of-Africa population size being 1861, and the migration rate between Africa and out-of-Africa being 1.5 × 10^−4^ (migrant proportion of population, per generation). The Asian and European populations split from the out-of-Africa population 920 generations ago, with sizes of 1032 for Europe and 554 for Asia, and growth rates of 3.8 × 10^−3^ for Europe and 4.8 × 10^−3^ for Asia (the African population size remained constant at 14474). Migration rates post-split were 2.5 × 10^−5^ between Africa and Europe, 7.8 × 10^−6^ between Africa and Asia, and 3.11 × 10^−5^ between Europe and Asia [18].

We create an admixed population with admixture occurring 12 generations ago. The admixed population had an initial size of 30,000 and grew at a rate of 5% per generation, with 1/6 of the population of African ancestry, 1/3 European, and 1/2 Asian.

We simulated sequence data with a mutation rate of 1.25 × 10^−8^ per basepair per meiosis, and a constant recombination rate of 1 × 10^−8^ per basepair per meiosis (i.e., 1 cM = 1 Mb). We simulated 500 individuals from the simulated admixed population, and 100 reference individuals from the three ancestral populations (Africa, Europe, and Asia). We simulated 30 chromosomes each of length 100 Mb. Our simulation code can be found in S1 Source Code.

After simulating the data, we removed all variants with minor allele frequency less than 5%, and 70% of the remaining variants. After removing these variants, 926,159 variants remained for analysis across the 30 simulated chromosomes, so that the data are similar to those on a 1M SNP array. We then added genotype error: 0.1% of genotypes were randomly chosen to have a random allele changed.

We followed the same analysis pipeline as for the real data, using RFMix [8] to infer local ancestry, Refined IBD [17] with a gap filling procedure to infer segments of identity by descent of length 2 cM and longer, and IBDNe [14] to estimate ancestry-specific effective population sizes. Further details are given below. The analysis pipeline can be found at http://faculty.washington.edu/sguy/asibdne/.

In addition, for the simulated data we needed to obtain the ground-truth effective population sizes for comparison with the estimated values. It is not straightforward to determine the populations’ effective sizes directly from the simulation parameters. So we simulated coalescence trees from the same simulation model as in the main simulation, and we utilized the known coalescence times and ancestral origins to determine the ground-truth ancestry-specific effective sizes. Specifically, we simulated 5000 replicate trees, each with 10,000 haplotypes sampled from the current-day admixed population. For each sampled haplotype, we determined its ancestral origin by tracing its path back through the tree to the first pre-admixture coalescence node. Considering a given ancestral population and a given generation (*g* < 100) before present, in simulated tree *i* we determined the number of branches, *b*_*i*_, that are ancestral to haplotypes derived from the given ancestral population. Any pair of branches could coalesce, so the number of opportunities for coalescence is *b*_*i*_ choose 2, or *b*_*i*_(*b*_*i*_ − 1)/2. We also determined the actual number of coalescences occurring in this generation for this ancestry population, *c*_*i*_. Combining results across the simulated trees, we obtained the conditional coalescence rate $\hat{q_{g}}=\left(\sum_{i=1}^{5000}c_{i}\right)/\left(\sum_{i=1}^{5000}b_{i}(b_{i}-1)/2\right)$. The large number of simulated trees (5000) and large number of sampled haplotypes per tree (10,000) result in an estimated $\hat{q_{g}}$ that is close to the true value *q*_*g*_. We then obtained the ancestry-specific effective size at generation *g* from the relationship *N*_*g*_ = 1/(2*q*_*g*_).

### Estimating IBD segments in heterogeneous data

In our previous development of IBDNe [14] we used IBDseq [27] to detect IBD segments because we were analyzing homogenous data and because assignment of an IBD segment to a particular haplotype was not necessary. However, IBDseq overestimates IBD segment lengths in heterogeneous data such as the admixed data analyzed in this study, because it does not account for linkage disequilibrium induced by population structure. The haplotype-based IBD detection method Refined IBD [17] is robust to genetic heterogeneity because the requirement that two haplotypes are identical over an extended region is very strong, and because haplotype frequencies estimated using appropriate approaches such as the Beagle model account for linkage disequilibrium. Further, Refined IBD assigns IBD segments to individual haplotypes which is necessary for determining the local ancestry within the IBD segment, whereas IBDseq does not assign phase to the IBD segments. We used the Beagle 4.1 version of Refined IBD with default settings unless otherwise noted, and we applied a 2 cM IBD segment length threshold.

In order to successfully apply IBDNe, we need an IBD detection method that has high power to detect IBD segments with length greater than a threshold (2 cM in this study), and that estimates the lengths of these segments accurately. Genotype errors and haplotype phase errors can result in gaps in the estimated IBD segments when using a haplotype-based approach such as Refined IBD. A single long IBD segment may be reported as two shorter segments with a gap between them. This leads to underestimation of IBD segment lengths. We developed a software tool merge-ibd-segments.jar (available from the Beagle Refined IBD website http://faculty.washington.edu/browning/refined-ibd.html) to fill these gaps between reported segments. In this study we filled a gap between two detected IBD segments if the length of the gap was less than 0.6 cM and there was at most one pair of genotypes inconsistent with IBD (e.g. opposite homozygotes) in the gap. This strategy produces estimates of IBD segment length that are reasonably accurate for segments > 2 cM length, even in admixed populations (S4 Fig).

### Estimation of local ancestry

We used RFMix [8] version 1.5.4 to estimate local ancestry in each data set, simulated and real. For the Health ABC data, we used 112 CEU (CEPH from Utah) and 147 YRI (Yoruba from Ibadan Nigeria) samples from Hapmap3 [28] as reference samples. For the HCHS/SOL data, we used reference data from 195 West Africans, 63 Amerindians, and 527 Europeans obtained from the 1000 Genomes project and the Human Genome Diversity Project as previously described [13].

We used the “PopPhased” option and “-n 5” argument as recommended in the RFMix documentation. RFMix requires phased input data, and we used the phased genotypes generated by Beagle in the Refined IBD analysis. RFMix performs some rephasing of the data while inferring local ancestry. In order to match the local ancestry haplotypes to the IBD segment haplotypes, we adjusted the phasing of the RFMix Viterbi local ancestry calls to match the genotype phasing used in the IBD segment detection. Moving along the chromosome for an individual, we checked whether the phase of heterozygous genotypes matched between the RFMix rephasing and the Beagle phasing for the individual. We inferred a switch in the RFMix phasing relative to the Beagle phasing whenever the relative phasing of consecutive heterozygous genotypes changed. These switches were then applied to the RFMix Viterbi local ancestry calls for the individual in order to make the phasing of the ancestry calls consistent with the phasing of the IBD segments. The local ancestry proportion for an IBD segment was then determined from the phase-adjusted local ancestry calls for the corresponding haplotypes, using the mean called local ancestry from the two haplotypes. For example, if 41% of the length of the IBD segment had called local ancestry 1 for the first haplotype, and 43% of the length of the IBD segment had called local ancestry 1 for the second haplotype, then we considered the IBD segment to have 42% ancestry 1. We investigated the concordance between the called local ancestry proportions for pairs of IBD haplotypes in the HCHS data. Considering one ancestry at a time, we record the proportion of that ancestry for each IBD haplotype, and calculate the correlation of these proportions between the pairs of IBD haplotypes. For African ancestry, we obtain a correlation of 0.980; for European ancestry, 0.982; for Native American ancestry, 0.987.

### Ancestry-specific effective population size estimation

An IBD segment may span a change in ancestry if the most recent common ancestor lived more recently than the commencement of admixture. The total length of an IBD segment provides information about the time to the most recent common ancestor, so one cannot simply cut the IBD segment into smaller ancestry-homogeneous segments. In order to estimate effective size, it is necessary to consider both the length of detected ancestry-specific IBD (which gives information about coalescence probabilities) and the length of the overall IBD segment (which gives information about the coalescence time). Specifically, the IBDNe program assigns each IBD segment fractionally to various coalescence times (measured in discrete numbers of generations) depending on the IBD segment length and the current estimates of effective population size history, and then uses the total (sum of fractional counts across IBD segments) for each generation to re-estimate the effective population size of that generation. For ancestry-specific effective size, we only want to consider pairs of haplotypes of the particular ancestry, that is those parts of IBD segments that are of that ancestry, but we need to use the full IBD segment lengths to estimate the coalescence times. One way to achieve this would be to weight the IBD segments by their proportion of the given ancestry. Implementing this approach would add complexity to the IBDNe program so instead we randomly assign the IBD segment to an ancestry based on the ancestry proportions of the segment. For example, if the segment has 80% of its length called as ancestry 1 and 20% as ancestry 2, we assign it to ancestry 1 with probability 80% and to ancestry 2 with probability 20%. This approach has the same average input as the fractional assignment approach, and results in an unbiased estimate of the observed total length of IBD corresponding to a particular ancestry.

### Ancestry-adjusted number of pairs of sampled haplotypes

The expected total length of IBD attributable to generation *g*, *E*_*g*_, is proportional to the number of pairs of haplotypes that can generate that IBD. Two haplotypes can only be IBD with respect to a given ancestry at genomic positions where both haplotypes have that ancestry. We assume the local ancestry of individuals *i* and *j* is independent, which is approximately true for all but close relatives. If individuals *i* and *j* have proportion *p*_*i*_ and *p*_*j*_ of the given ancestry, respectively, then the expected proportion of genome over which a random haplotype drawn from individual *i* and a random haplotype drawn from individual *j* both have that ancestry is *p*_*i*_*p*_*j*_. If there are *n* individuals, then the ancestry-adjusted number of pairs of haplotypes is $\sum_{i=1}^{n-1}\sum_{j=i+1}^{n}4p_{i}p_{j}$.

### Removal of relative pairs

Estimation of effective population size assumes a random population sample. Non-random ascertainment can affect the estimated effective size for the first few generations before present. In particular, an excess of close relatives in a data set will result in an excess of very large IBD segments, and hence a downward-biased estimate of the most recent effective size. By default, the IBDNe program searches for close relatives (half-sibs and closer) by looking for high genome-wide levels of IBD sharing and removes such pairs from consideration [14]. In the ancestry-specific case, the total proportion of the genome that could be IBD for the specific ancestry will tend to be low, and hence IBDNe won’t identify the pairs. We therefore identify pairs of close relatives using all IBD segments, and manually remove pairs with over 1200 cM of IBD genome-wide from the ancestry-specific analysis by removing the corresponding IBD segments and removing the pairs from the calculation of the ancestry-adjusted numbers of pairs of sampled haplotypes.

### Bootstrap confidence intervals

We obtain confidence intervals for the estimated effective population size trajectories by bootstrap resampling of chromosomes. The bootstrapping is performed by the IBDNe program. Each bootstrap replicate resamples from the chromosomes with replacement to obtain the same number of chromosomes (22 for autosomal human data) as in the original data. The program estimates the effective population size trajectory for each bootstrap replicate in the same manner as for the original data. We use the default number of bootstrap replicates (80) and show the 2.5^th^ and 97.5^th^ percentiles. These intervals provide an approximate measure of the precision of the effective population size estimates.

### Improvements to the IBDNe algorithm

In the course of this project, we made some changes to the IBDNe software to simplify the algorithm and reduce computation time.

The original version of the IBDNe program performs 50 runs of an iterated method of moments algorithm and reports the harmonic mean of the population size at each generation from the 50 runs. Each run starts with a random initial effective population size at each generation before the present (a trajectory) and updates this estimate at each iteration for 50 iterations. In each iteration, the algorithm fits a piecewise exponential growth curve, with a new growth rate every 8 generations. For each run, the first change in growth rate is randomly chosen to occur between 5 and 12 (inclusive) generations before the present [14].

The revised IBDNe program performs 8 runs of the iterated method of moments algorithm and uses a fixed initial effective size trajectory for each run (a constant effective population size of 100). As before the algorithm fits a piecewise exponential growth curve, with a new growth rate every 8 generations. The first change in growth rate still occurs between generations 5 and 12 (inclusive), and each of the 8 runs uses a different first time of change in growth rate. As before the program averages results from runs of the algorithm using a harmonic mean. We increased the default number of iterations per run from 50 to 1000, as we found continuing improvements in fit up to this point. These changes are implemented in the revised release of the IBDNe program (version 1.1). The program can be freely downloaded from http://faculty.washington.edu/browning/ibdne.html.

To allow ancestry-specific estimation of effective population size, we further modified IBDNe to include a parameter that specifies the ancestry-adjusted number of pairs of sampled haplotypes (see **Ancestry-adjusted number of pairs of sampled haplotypes)**. If this parameter is missing, IBDNe will assume that overall (rather than ancestry-specific) effective size is being estimated, and determine the number of sampled haplotype pairs directly from the data. Thus one can give IBDNe ancestry-specific IBD segments and an ancestry-adjusted number of pairs of sampled haplotypes (both of which are obtained using the procedures described above) in order to obtain ancestry-specific estimates of effective population size.

### HCHS/SOL data

HCHS/SOL is a study of 16,415 self-identified Hispanic/Latino individuals aged 18–74 (mean 41), with baseline examination in 2008–2011. The individuals were sampled by household in four US field centers (Chicago, IL; Miami, FL, Bronx, NY; San Diego, CA). The individuals were genotyped on an Illumina Omni 2.5M array with additional custom content, and the genotype data and phenotype data for 12,437 individuals are posted on dbGaP (accession numbers phs000880.v1.p1 and phs000810.v1.p1). In our IBDNe analysis, we excluded data from individuals who were not included in the dbGaP posting.

Segments of IBD were called with Beagle version 4 (r1203) [17] using genotyped SNPs with minor allele frequency > 0.02. Local ancestry calls were made previously with RFMix [13]. In analysis of effective population size, we considered only individuals whose four grandparents all had the same country of origin. Countries with fewer than 120 sampled individuals were excluded. Sample sizes by country can be found in Table 1. Due to the household-based sampling design, the data include many close relatives. We excluded IBD from close relative pairs as described above. We used the HapMap recombination map [29] for analyses with RFMix, for analyses with Beagle, and to determine IBD segment lengths.

### Health ABC data

Individuals from the Healthy Aging and Body Composition (Health ABC) study were genotyped for the CIDR Visceral Adiposity Study. Genotype data were obtained for around 600 self-identified black and 800 self-identified white individuals (here referred to as African American and European American, respectively) from each of Memphis and Pittsburgh (Table 2). All individuals were 70–79 years old at recruitment in 1997–1998. Participants were identified from a random sample of white Medicare beneficiaries and all age-eligible community-dwelling black residents in designated zip code areas surrounding the field centers in Pittsburgh, Pennsylvania, and Memphis, Tennessee [30]. Genotype data from an Illumina Human1M-Duo BeadChip array were downloaded from dbGaP (study accession phs000169.v1.p1). We removed SNPs with call rate <99% in either the African American or the European American samples, Hardy-Weinberg equilibrium p-value < 10^−6^ in either the African American or the European American samples, or minor allele frequency < 1% in either the African American or the European American samples.

We called segments of IBD using Beagle 4.1. We used HapMap phase 3 CEU and YRI samples (Utah residents with ancestry from northern and western Europe and Yoruba in Ibadan, Nigeria respectively) [28] as reference panels for the local ancestry calling, utilizing SNPs genotyped in both the HapMap phase 3 and Health ABC data. We phased the reference HapMap data using the phased Health ABC data from our Beagle Refined IBD analysis as a phasing reference panel.

We used the HapMap recombination map [29] for analyses with RFMix, for analyses with Beagle, and to determine IBD segment lengths. The Health ABC data did not contain any close relatives.

## Supporting information

S1 Fig Simulation with merging populations. The simulation model is the same as for the main simulation (see Methods) prior to admixture. Admixture occurred 12 generations ago with the three populations merging; the admixed population size at admixture was the sum of the sizes of the three contributing populations. The admixed population grew at a rate of 5% per generation. The figure shows analysis of 500 simulated individuals from the admixed population. Each column is one of the three simulated ancestries. The y-axes show ancestry-specific effective population size (*N*_*e*_), plotted on a log scale. The x-axes show generations before present. The dashed lines show simulated effective population sizes. The solid black lines show estimated ancestry-specific effective population sizes, and the gray regions show 95% bootstrap confidence intervals.
(TIF)

S2 Fig Simulation with continuous admixture. The simulation model is the same as for the main simulation (see Methods) prior to admixture. Admixture began to occur 20 generations ago, with Europeans migrating into Asia at a rate of 1% per generation and Africans migrating into Asia at a rate of 1% per generation. That is, the number of new immigrants in the admixed Asian population each generation was 2% of the total population size, with half of these immigrants being from Europe and half from Africa. The admixed population grew at a rate of 5% per generation. The figure shows analysis of 500 simulated individuals from the admixed population. Each column is one of the three simulated ancestries. The y-axes show ancestry-specific effective population size (*N*_*e*_), plotted on a log scale. The x-axes show generations before present. The dashed lines show simulated effective population sizes. The solid black lines show estimated ancestry-specific effective population sizes, and the gray regions show 95% bootstrap confidence intervals.
(TIF)

S3 Fig Simulation with population structure. The simulation model is the same as for the main simulation (see Methods) with the only difference being that immediately after admixture the admixed population split into two equally sized sub-populations. During the post-admixture generations, these two sub-populations exchanged migrants at a rate of 10% per generation. We analyzed an unbiased sample in which 50% of the sampled individuals are from each of the two sub-population (A; top row), and a biased sample in which 90% of the sampled individuals are from one of the two sub-populations (B; bottom row). In each case 500 simulated individuals from the admixed population were analyzed. Each column is one of the three simulated ancestries. The y-axes show ancestry-specific effective population size (*N*_*e*_), plotted on a log scale. The x-axes show generations before present. The dashed lines show simulated effective population sizes (ignoring the post-admixture population structure). The solid black lines show estimated ancestry-specific effective population sizes, and the gray regions show 95% bootstrap confidence intervals.
(TIFF)

S4 Fig Hexbin plot of accuracy of IBD length inferred with Refined IBD, with a gapfill procedure, for simulated admixed data. Segments with true or inferred length > 6 cM, or with true and inferred length both < 2 cM are omitted. In determination of true segments, only those segments with length > 1 cM are considered, and in inference of IBD segments, only those with inferred length > 1cM are considered; segments with true length > 2 cM with no corresponding inferred IBD segment are shown as having an inferred IBD segment length of 0, and segments with inferred IBD length > 2 cM that do not correspond to a true IBD segment are shown as having a true IBD length of 0. The plotting region is divided into small hexagons, and the color of a hexagon represents the count of the number of segments falling into it (black for many segments, white for zero or very few segments).
(TIF)

S1 Source Code Msprime code used to simulate data. (PDF)

S1 Table Average total length (in cM) of detected IBD segments for unrelated pairs of individuals within each population. (PDF)
