# Supplementary figures and images for: Ancestry-specific recent effective population size in the Americas
Source: PLoS Genet. 2018 May 24;14(5):e1007385. doi: 10.1371/journal.pgen.1007385 (PMC5967706; doi:10.1371/journal.pgen.1007385)

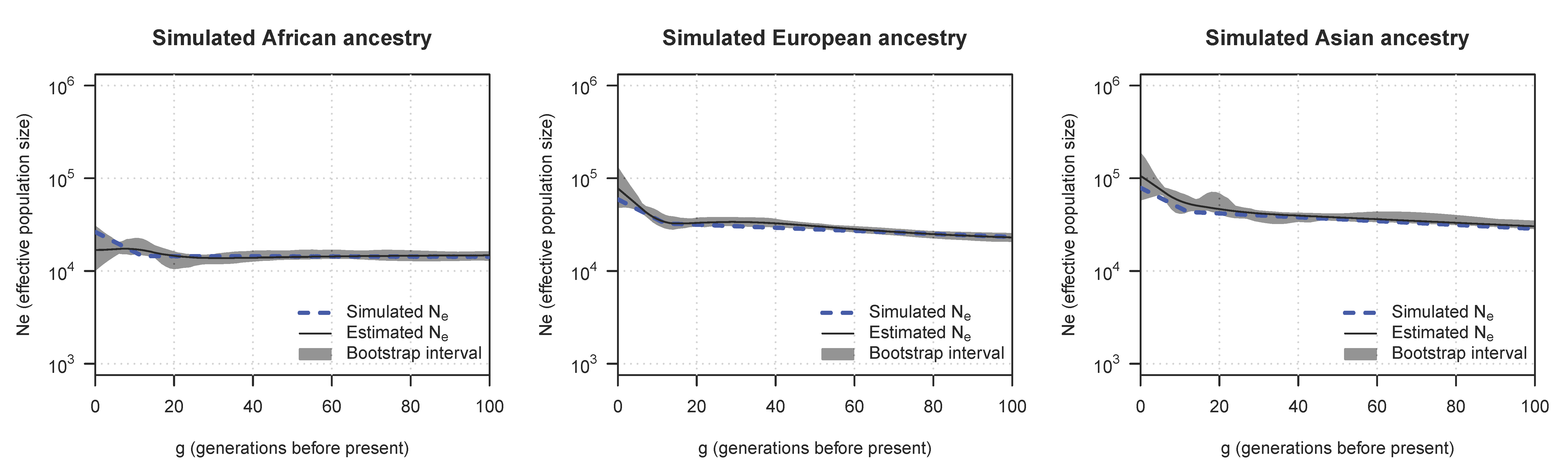

Supplement: S1 Fig — The simulation model is the same as for the main simulation (see Methods) prior to admixture. Admixture occurred 12 generations ago with the three populations merging; the admixed population size at admixture was the sum of the sizes of the three contributing populations. The admixed population grew at a rate of 5% per generation. The figure shows analysis of 500 simulated individuals from the admixed population. Each column is one of the three simulated ancestries. The y-axes show ancestry-specific effective population size (Ne), plotted on a log scale. The x-axes show generations before present. The dashed lines show simulated effective population sizes. The solid black lines show estimated ancestry-specific effective population sizes, and the gray regions show 95% bootstrap confidence intervals. (TIF) [file pgen.1007385.s001.tif]

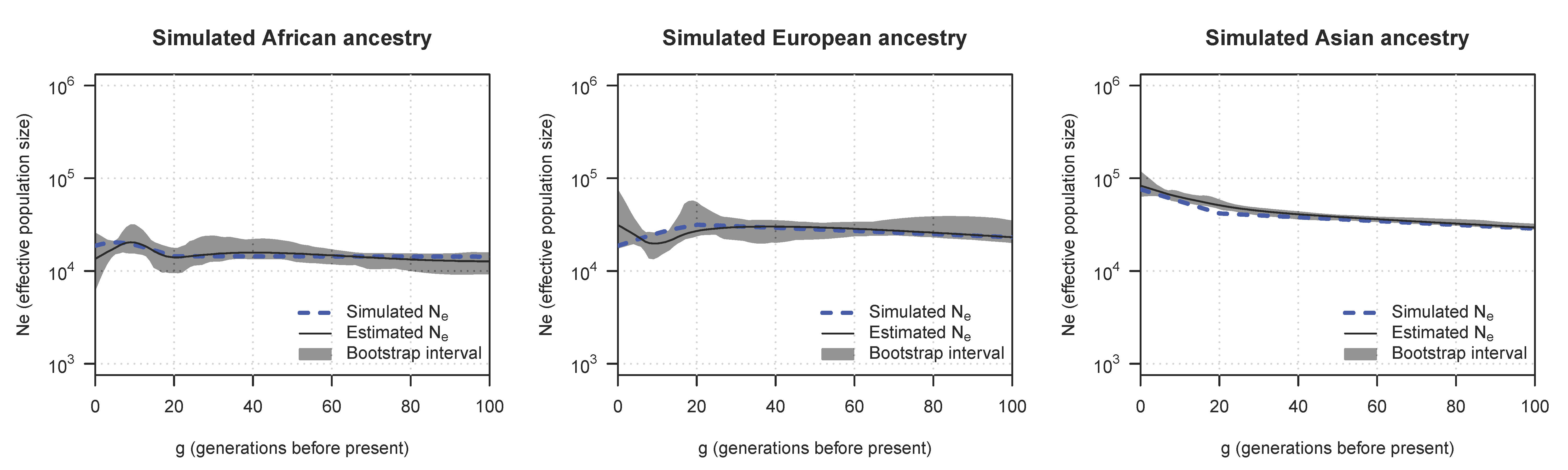

Supplement: S2 Fig — The simulation model is the same as for the main simulation (see Methods) prior to admixture. Admixture began to occur 20 generations ago, with Europeans migrating into Asia at a rate of 1% per generation and Africans migrating into Asia at a rate of 1% per generation. That is, the number of new immigrants in the admixed Asian population each generation was 2% of the total population size, with half of these immigrants being from Europe and half from Africa. The admixed population grew at a rate of 5% per generation. The figure shows analysis of 500 simulated individuals from the admixed population. Each column is one of the three simulated ancestries. The y-axes show ancestry-specific effective population size (Ne), plotted on a log scale. The x-axes show generations before present. The dashed lines show simulated effective population sizes. The solid black lines show estimated ancestry-specific effective population sizes, and the gray regions show 95% bootstrap confidence intervals. (TIF) [file pgen.1007385.s002.tif]

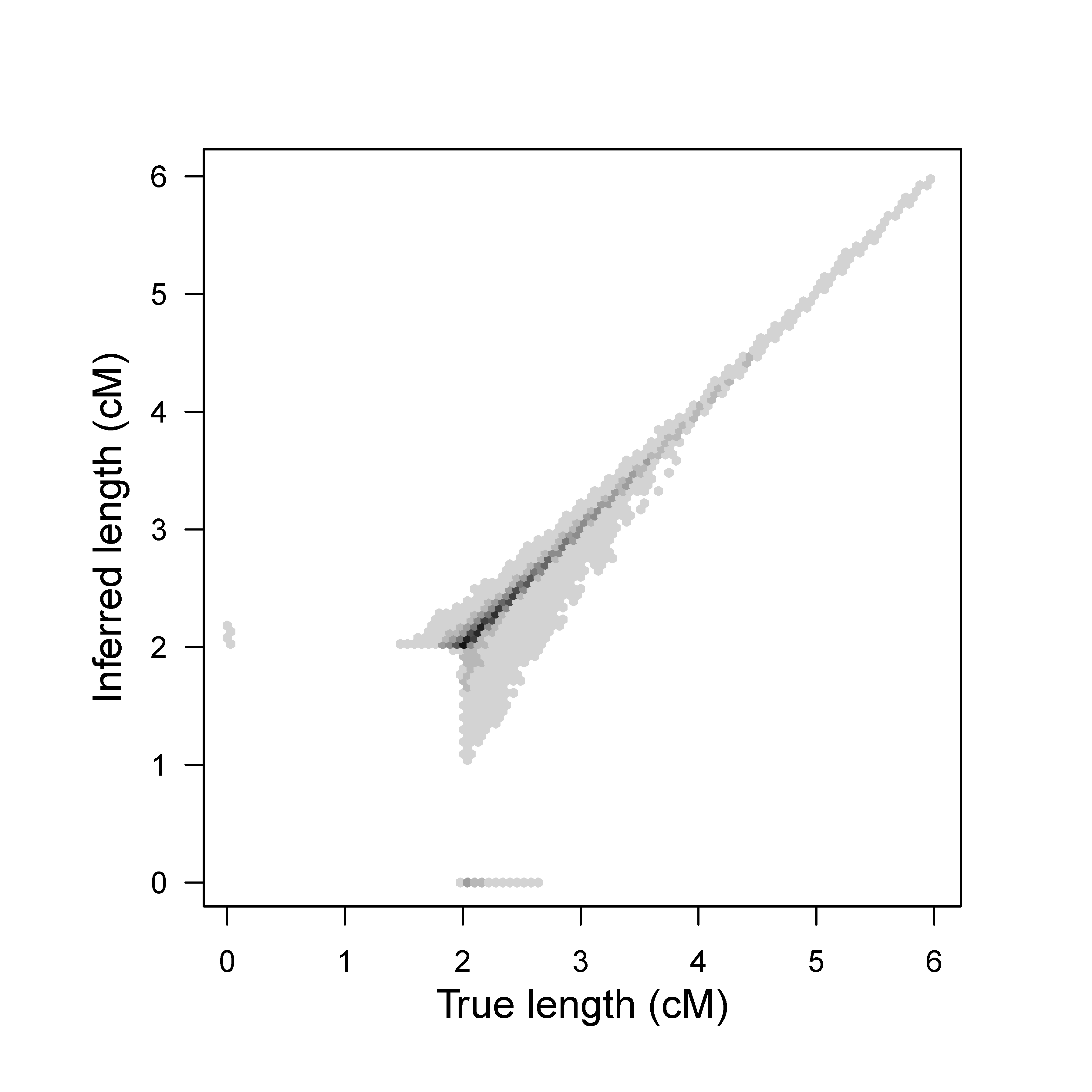

Supplement: S4 Fig — Segments with true or inferred length > 6 cM, or with true and inferred length both < 2 cM are omitted. In determination of true segments, only those segments with length > 1 cM are considered, and in inference of IBD segments, only those with inferred length > 1cM are considered; segments with true length > 2 cM with no corresponding inferred IBD segment are shown as having an inferred IBD segment length of 0, and segments with inferred IBD length > 2 cM that do not correspond to a true IBD segment are shown as having a true IBD length of 0. The plotting region is divided into small hexagons, and the color of a hexagon represents the count of the number of segments falling into it (black for many segments, white for zero or very few segments). (TIF) [file pgen.1007385.s004.tif]
