# Supplementary material for: Ancestry-specific recent effective population size in the Americas
Source: PLoS Genet. 2018 May 24;14(5):e1007385. doi: 10.1371/journal.pgen.1007385 (PMC5967706; doi:10.1371/journal.pgen.1007385)
Supplement: S1 Source Code — (PDF) [file pgen.1007385.s005.pdf]

**S1 Source Code. Msprime code used to simulate data.**

```
import msprime, sys
from math import log
from math import exp
seed = int(sys.argv[1])

mu=1.25e-8 # mutation rate per bp
rho=1e-8 # recombination rate per bp
nbp = 1e8 # generate 100 Mb
N0=7310 # initial population size
Thum=5920 # time (gens) of advent of modern humans
Naf=14474 # size of african population
Tooa=2040 # number of generations back to Out of Africa
Nb=1861 # size of out of Africa population
mafb=1.5e-4 # migration rate Africa and Out-of-Africa
Teu=920 # number generations back to Asia-Europe split
Neu=1032; Nas=554 # bottleneck population sizes
mafeu=2.5e-5; mafas=7.8e-6; meuas=3.11e-5 # mig. rates
reu=0.0038 # growth rate per generation in Europe
ras=0.0048 # growth rate per generation in Asia
Tadmix=12 # time of admixture
Nadmix=30000 # initial size of admixed population
radmix=.05 # growth rate of admixed population

# pop0 is Africa, pop1 is Europe, pop2 is Asia, pop3 is admixed
refsamplesize = 200
admsamplesize = 1000
pop_config = [
msprime.PopulationConfiguration(sample_size=refsamplesize,initial_size=Naf,growth_rate=0.0),
msprime.PopulationConfiguration(sample_size=refsamplesize,initial_size=Neu*exp(reu*Teu),growth_rate=reu),
msprime.PopulationConfiguration(sample_size=refsamplesize,initial_size=Nas*exp(ras*Teu),growth_rate=ras),
msprime.PopulationConfiguration(sample_size=admsamplesize,initial_size=Nadmix*exp(radmix*Tadmix),growth_rate=radmix)]
mig_mat = [[0,mafeu,mafas,0],[mafeu,0,meuas,0], [mafas,meuas,0,0],[0,0,0,0]]

# Admixture event, 1/6 Africa, 2/6 Europe, 3/6 Asia
admixture_event = [  msprime.MassMigration(time=Tadmix,source=3,destination=0,proportion=1.0/6.0),
msprime.MassMigration(time=Tadmix+0.0001,source=3,destination=1,proportion=2.0/5.0),
msprime.MassMigration(time=Tadmix+0.0002,source=3,destination=2,proportion=1.0)]

# Asia and Europe split
eu_event = [
msprime.MigrationRateChange(time=Teu,rate=0.0),
msprime.MassMigration(time=Teu+0.0001,source=2,destination=1,proportion=1.0),
msprime.PopulationParametersChange(time=Teu+0.0002,initial_size=Nb,growth_rate=0.0,population_id=1),
msprime.MigrationRateChange(time=Teu+0.0003,rate=mafb,matrix_index=(0,1)),
msprime.MigrationRateChange(time=Teu+0.0003,rate=mafb,matrix_index=(1,0))]

# Out of Africa event
ooa_event = [
msprime.MigrationRateChange(time=Tooa,rate=0.0),
msprime.MassMigration(time=Tooa+0.0001,source=1,destination=0,proportion=1.0)]

# initial population size
init_event = [    msprime.PopulationParametersChange(time=Thum,initial_size=N0,population_id=0)]
```

```
events = admixture_event + eu_event + ooa_event + init_event

treeseq = msprime.simulate(population_configurations=pop_config, migration_matrix=mig_mat, demographic_events=events, length=nbp, recombination_rate=rho, mutation_rate=mu, random_seed=seed)
```
