# Supplementary material for: Ancestry-specific recent effective population size in the Americas
Source: PLoS Genet. 2018 May 24;14(5):e1007385. doi: 10.1371/journal.pgen.1007385 (PMC5967706; doi:10.1371/journal.pgen.1007385)
Supplement: S1 Table — (PDF) [file pgen.1007385.s006.pdf]

Population[a]	2-3cM[b]	3-5cM	5-10cM	10+cM	Total[c]
**Colombia**	1.9	1.5	2.4	1.7	7.6
**Cuba**	1.1	0.9	1.1	0.7	3.7
**Dominican Republic**	1.2	1.6	3.3	3.1	9.2
**Ecuador**	1.9	1.4	1.8	1.4	6.5
**Guatemala**	1.4	1.4	2.1	1.6	6.6
**Honduras**	1.7	1.9	3.5	2.6	9.7
**Mexico**	1.2	0.8	0.7	0.3	3.0
**Nicaragua**	2.4	1.9	2.8	2.1	9.2
**Puerto Rico**	3.6	6.7	14.8	10.0	35.1
**Memphis AA**	1.5	0.4	0.2	0.2	2.3
**Memphis EA**	2.0	0.6	0.3	0.1	2.9
**Pittsburgh AA**	1.3	0.3	0.2	0.2	2.0
**Pittsburgh EA**	2.7	2.6	2.0	0.3	7.5

[a] Population is country of origin of grandparents for the HCHS/SOL populations (first nine populations in table). For the Health ABC populations (last four populations in table), EA is European American, AA is African American.

[b] Average total length of detected IBD in cM for segments with inferred length in the specified range, per pair of unrelated individuals in the sample from the population.

[c] Average total length of detected IBD in cM for all segments with inferred length ≥ 2 cM, per pair of unrelated individuals in the sample from the population.
